# Synthesis and hydrogenation application of Pt–Pd bimetallic nanocatalysts stabilized by macrocycle-modified dendrimer

**DOI:** 10.1098/rsos.171414

**Published:** 2017-12-13

**Authors:** Zhijun Jin, Haiyan Xiao, Wei Zhou, Dongqiao Zhang, Xiaohong Peng

**Affiliations:** Department of Polymer Science and Engineering, South China University of Technology, Guangzhou 510640, People's Republic of China

**Keywords:** poly(propylene imine) dendrimer, triolefinic macrocycle, catalytic hydrogenation, Pt–Pd bimetallic nanoparticle, nitrile butadiene rubber, dimeric acid

## Abstract

Different generations of poly(propylene imine) (G*_n_*-PPI) terminated with N-containing 15-membered triolefinic macrocycle (G*_n_*M) (*n* = 2, 3, 4, 5) were prepared. The bimetallic nanoparticle catalysts G*_n_*M-(Pt*_x_*/Pd_10−*x*_) (*x* = 0, 3, 5, 7, 10) were prepared by the synchronous ligand-exchange reaction between G*_n_*M and the complexes of Pt(PPh_3_)_4_ and Pd(PPh_3_)_4_. The structure and catalytic properties of G*_n_*M-(Pt*_x_*/Pd_10−*x*_) were characterized via Fourier transform infrared spectroscopy, ^1^H nuclear magnetic resonance spectroscopy, X-ray diffraction, X-ray photoelectron spectroscopy, high-resolution transmission electron microscopy, energy-dispersive spectroscopy and inductively coupled plasma atomic emission spectroscopy. The novel bimetallic Pd–Pt nanoparticle catalysts stabilized by dendrimers (DSNs) present higher catalytic activities for the hydrogenation of dimeric acid (DA) than that of nitrile butadiene rubber (NBR). It can be concluded that bimetallic Pd–Pt DSNs possess alloying and synergistic electronic effects on account of the hydrogenation degree (HD) of DA and NBR. Furthermore, the HD of DA and NBR shows a remarkable decrease with the incremental generations (*n*) of G*_n_*M-(Pt_3_/Pd_7_) (*n* = 2, 3, 4, 5).

## 1. Introduction

For the past few years, bimetallic nanoparticles have attracted widespread attention among the academic community owing to their applications in fine chemicals, nanomedicine and petrochemical technology [1–4].

As an oil chemical product with low toxicity, extensive sources and renewable materials, dimeric acid (DA) is widely applied to the synthesis of novel polymeric materials.

Hydrogenated dimeric acid (HDA) is produced by unsaturated DA through catalytic hydrogenation reaction. HDA has better thermostability and machinability than unsaturated DA, which is widely used in the synthesis of polyamides, polyesters, mineral oil additives, surface active agents and waterproofing agents [5–7].

Compared with nitrile butadiene rubber (NBR), hydrogenated nitrile butadiene rubber (HNBR) possesses physicochemical properties of thermostability, inoxidizability and high oil durability, due to its lower ratio of unsaturated carbon–carbon bonds than NBR [8]. Nowadays, the solution hydrogenation of NBR has been the main technical route for preparing HNBR hydrogenated by homogeneous or heterogeneous catalysts. To contrast the difference between the two types of catalysts, the former possesses higher catalytic activity and leads to higher degree of hydrogenation, which attracts extensive attention for the catalytic hydrogenation of NBR. However, their high cost as well as difficult catalyst removal hinders their application in HNBR production. Accordingly, extensive research and development work has been conducted on novel hydrogenation catalysts for NBR, aimed at obtaining high selectivity, efficient catalytic activity, cost-effectiveness and ease of removal [9].

In comparison to monometallic nanoparticles, bimetallic nanoparticles have unique properties, such as synergistic electric effect, high selectivity, efficient catalytic activity and cost-effectiveness [10–13]. Among these bimetallic nanoparticles, supported palladium–metal (Pd-M) bimetallic nanoparticle catalysts have attracted considerable attention because of their remarkable activity and high selectivity [14–17]. It is worth noting that the catalytic activity of Pd-M nanoparticle catalysts could be further enhanced by developing novel preparation strategies, selecting suitable support materials and introducing a second or even third ingredient to form bimetallic or multimetallic nanoparticles [15,18]. The methods of bimetallic nanoparticles synthesized in the template of dendrimer (DSNs) have been extensively studied towards their applications in catalytic hydrogenation. For example, Chung & Rhee [12] prepared Pd–Rh bimetallic nanoparticles in the presence of poly(amidoamine) dendrimers with surface hydroxyl groups for the selective partial hydrogenation of 1,3-cyclooctadiene.

A dendrimer has a controlled molecular weight, well-defined structure, high symmetry of geometric structure, nanoscale of molecular size and surface modification [13]. Therefore, it can act as an ideal template to control the morphology and size of dendrimer-encapsulated metal nanoparticle catalysts. Moreover, the DSNs can be prepared when transferring the catalytic active centres to the surface of the end-functionalized dendrimer [19].

The 15-membered triolefinic macrocycle (MAC), possessing excellent oxidation-resistant property and insolubility of alcohols, is a suitable electron donor for transition metals and easily removed from the reaction system [20]. Therefore, bimetallic DSNs with high stability, easy-removal and favourable catalytic activity and selectivity can be obtained [21], which will be a promising research field for the catalytic hydrogenation of unsaturated compounds.

In this paper, G*_n_*M-(Pt*_x_*/Pd_10−*x*_) (*n* = 2, 3, 4, 5) DSN catalysts were prepared by the synchronous ligand-exchange reaction between precursors (Pt(PPh_3_)_4_, Pd(PPh_3_)_4_) and MAC-terminated G*_n_*-PPI (G*_n_*M) (*n* = 2, 3, 4, 5) as the template. The hydrogenation of unsaturated compounds was carried out to evaluate the catalytic ability and selectivity of bimetallic DSN catalysts. It can be concluded that bimetallic Pd–Pt DSNs possess alloying and synergistic electronic effects. Furthermore, the HD of DA and NBR shows a remarkable reduction with the incremental generations (*n*) of G*_n_*M-(Pt_3_/Pd_7_).

## 2. Experimental set-up

### 2.1. Materials

Pt(PPh_3_)_4_ (99 wt%), Pd(PPh_3_)_4_ (99 wt%) and triphenylphosphine (99 wt%) were obtained from the Shanghai Macklin Biochemical Co. Ltd. Poly(propylene imine) dendrimers (G*_n_*-PPI) with a diaminobutane core were purchased from SyMO-Chem BV (Eindoven University of Technology, The Netherlands). Nitrogen-containing MAC was synthesized in our laboratory as described in the literature [13]. DA (98 wt%, *M*_w_ = 300.2) was purchased from Fujian Liancheng Baixin Science and Technology Co. Ltd. NBR (N31, ACN = 33.5 wt%, *M*_w_ = 350 000) was purchased from ZEON Corporation in Japan. All other reagents and solvents were of analytical grade and used without further purification.

### 2.2. Synthesis of MAC-terminated G*_n_*-PPI (G*_n_*M)

**Scheme 1.**
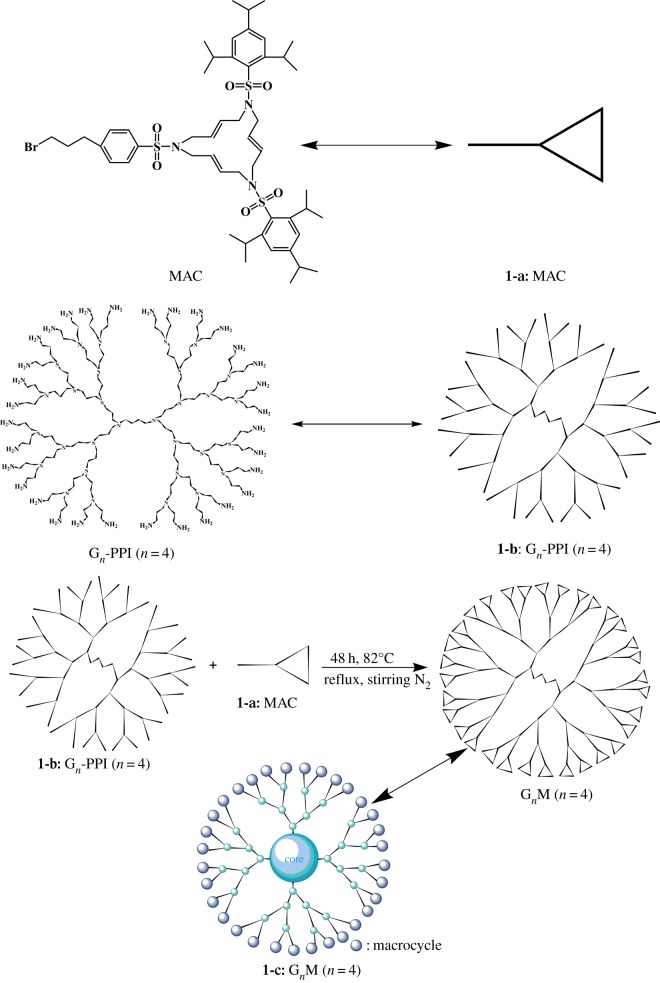
The synthesis route of G*_n_*M. MAC, G*_n_*-PPI, and G*_n_*M are simplified as **1-a**, **1-b** and **1-c**, respectively.

G_2_-PPI (0.1083 g, 0.15 mmol)/MAC (1.200 g, 1.2 mmol), G_3_-PPI (0.1263 g, 0.075 mmol)/MAC (1.200 g, 1.2 mmol), G_4_-PPI (0.1316 g, 0.0375 mmol)/MAC (1.200 g, 1.2 mmol) and G_5_-PPI (0.1346 g, 0.01875 mmol)/MAC (1.200 g, 1.2 mmol) were added to K_2_CO_3_ (0.4800 g, 3.5 mmol) and KI (1.200 g, 0.72 mmol) in KCN (40 ml). The mixture was stirred for 48 h at 82°C under the protection of nitrogen. The residual salts were eliminated by filtration and the filtered solution was evaporated. The residue was washed three times with ethyl acetate–pentane (6 : 1 v/v) and dried to afford G*_n_*M as white solid. The synthesis route is shown in scheme 1.

### 2.3. Preparation of dendrimer-stabilized G*_n_*M-(Pt*_x_*/Pd_10−*x*_) bimetallic nanoparticles

**Scheme 2.**
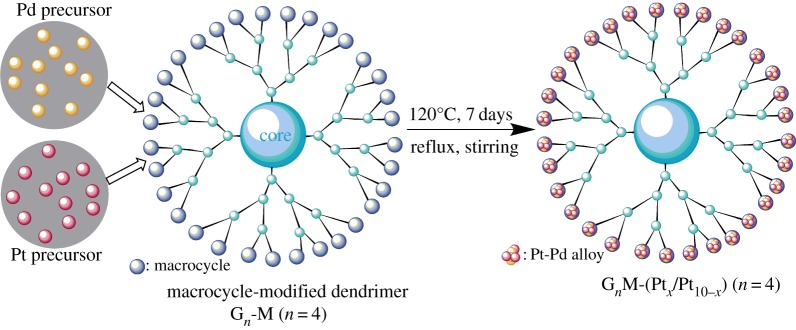
The synthesis route of G*_n_*M-(Pt*_x_*/Pd_10−*x*_) (*n* = 2, 3, 4, 5).

Dendrimer-stabilized bimetallic nanoparticles were prepared via a simultaneous co-complexation method of complexes of Pt(PPh_3_)_4_ and Pd(PPh_3_)_4_ (Pt : Pd = 10 : 0, 7 : 3, 5 : 5, 3 : 7, 0 : 10 mol), and quantitative G*_n_*M (total complexes: G*_n_*M = 2*^n^*: 1 mol) which were dissolved in dimethylformamide (25 ml), and the mixture was refluxed with stirring for 7 days at 140°C under nitrogen (scheme 2). Furthermore, the solvent was removed under reduced pressure, and the residue was separated with *n*-pentane–tetrahydrofuran (10 : 1, v/v) to afford the brown G*_n_*M-(Pt*_x_*/Pd_10−*x*_) bimetallic nanoparticles.

### 2.4. Hydrogenation of the dimeric acid and nitrile-butadiene rubber

**Scheme 3.**
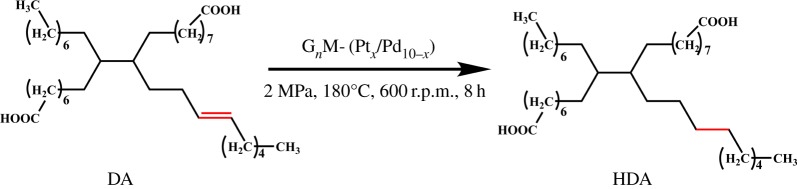
The hydrogenation of DA catalyzed by G*_n_*M-(Pt*_x_*/Pd_10−*x*_).

The hydrogenation of DA was carried out in the presence of bimetallic catalysts in xylene at 180°C under hydrogen (scheme 3). Xylene (30 ml), 1.8 g DA and a certain amount of G_4_M-(Pt*_x_*/Pd_10−*x*_) (catalyst/DA = 0.45 wt%) were added to a 50 ml high-pressure reactor. The hydrogen gas was introduced into the reactor and maintained at 2.0 MPa after the reactor was degassed using hydrogen for six times at room temperature. The reaction system was maintained for 8 h at 180°C with an agitating speed of 600 r.p.m. The system was cooled down. The mixture was evaporated to obtain viscous liquid; then, G*_n_*M-(Pt*_X_*/Pd_10−*X*_) was precipitated by adding *n*-pentane (60 ml). Finally, the precipitate was separated and the filtrate was dried to obtain HDA.

**Scheme 4.**
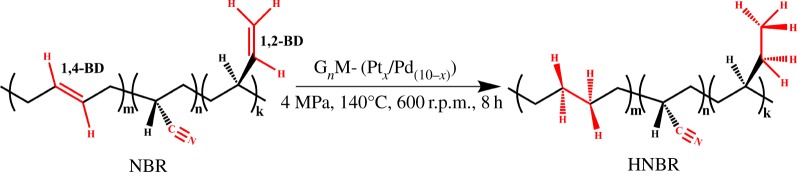
The hydrogenation of NBR catalyzed by G*_n_*M-(Pt*_x_*/Pd_10−*x*_).

The hydrogenation of NBR was carried out in the presence of bimetallic catalysts in xylene at 140°C under hydrogen (scheme 4). Xylene (30 ml), 1.5 g HNBR and a certain amount of G*_n_*M-(Pt*_x_*/Pd_10−*x*_) (catalyst/DA = 0.45 wt%) were added to a 50 ml high-pressure reactor. The hydrogen gas was introduced into the reactor and maintained at 4.0 MPa after the mixture was degassed using hydrogen for six times at room temperature. The reaction system was maintained for 8 h at 140°C with an agitating speed of 600 r.p.m. After the completion of the reaction, the system was cooled down. Then HNBR was obtained by unloading the crude product, adding methanol to flocculate the rubber and drying flocculate for 6 h at 90°C.

### 2.5. Characterization

MAC, G*_n_*-PPI, G*_n_*M and G*_n_*M-(Pt*_x_*/Pd_10−*x*_) (*n* = 2, 3, 4, 5) were characterized by ^1^H nuclear magnetic resonance spectroscopy (^1^H NMR, Bruker Avance III HD, 600 MHz, CDCl_3_) and Fourier transform infrared spectroscopy (FTIR, Bruker Vertex 70). The degree of hydrogenation of HDA and NBR was recorded via FTIR. X-ray diffraction (XRD) data were measured using an X-ray diffractometer (D8 Advance, anode material: Cu, step size: 0.013°). Transmission electron microscopy (TEM) was carried out using a JEM-2100F (Jeol, Japan) instrument equipped with an energy-dispersive spectroscopy (EDS) detector. X-ray photoelectron spectroscopy (XPS) was conducted using a Kratos Axis Ultra DLD of British Kratos company. The inductively coupled plasma atomic emission spectroscopy (ICP-OES) data were obtained by a PE Optima8300.

The hydrogenation degree (HD) of DA was calculated by iodine number (*w*) referring to ASTM D 4607-94 [22]:

$$\text{HD}=\left(1-\frac{w}{w^{0}}\right)\times 100\%,$$

where *w* is the iodine number of DA and *w*^0^ is the iodine number of HDA.

The HD of NBR was obtained from [23]:

$$\text{HD}=\left(1-\frac{{A^{\prime }}_{970+917}/{A^{\prime }}_{2236}}{A_{970+917}/A_{2236}}\right)\times 100\%.$$

The peaks at 970, 917 and 2236 cm^−1^ are ascribed to *trans*-1,4-ethenyl group (1,4-BD) out-of-plane bending vibration, 1,2-ethenyl group (1,2-BD) out-of-plane bending vibration and nitrile group (C≡N) stretching vibration, respectively, as shown in scheme 4. $A^{\prime }$ is the peak area of the correlative characteristic peaks for HNBR in the FTIR absorption spectra, while *A* is the peak area of the correlative characteristic peaks for NBR in the FTIR absorption spectra. HD calculated by FTIR is based on the internal standard peak of nitrile group (C≡N; scheme 4).

## 3. Results and discussion

### 3.1. MAC-terminated G*_n_*-PPI (G*_n_*M)

**Figure 1.**
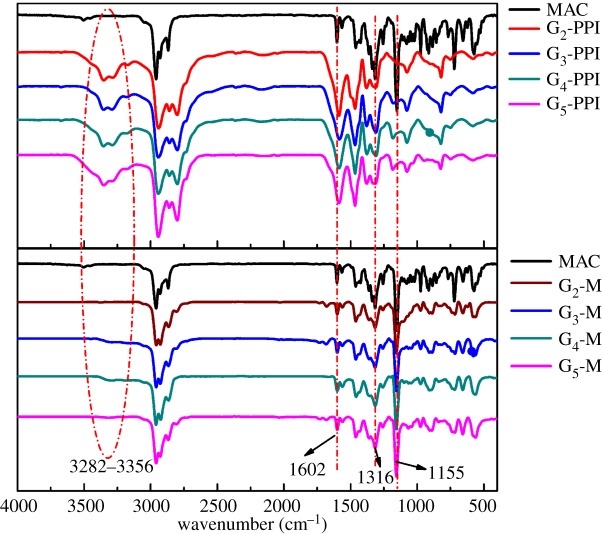
FTIR spectra of MAC, G*_n_*-PPI and G*_n_*M (*n* = 2, 3, 4, 5).

Figure 1 presents the changes in the FTIR absorption spectra of MAC, G*_n_* and G*_n_*M (*n* = 2, 3, 4, 5). The characteristic peaks of MAC, G*_n_* and G*_n_*M are at 1155 cm^−1^, 1316 cm^−1^ (O=S=O) and 1602 cm^−1^ (C=C), respectively, while the characteristic peaks of G*_n_*-PPI are at both 3282 and 3356 cm^−1^ attributed to symmetric and asymmetric stretching vibrations of –NH_2_ in G*_n_*-PPI, respectively. Compared with G*_n_*-PPI, the disappearance of two characteristic peaks of the 3282 and 3356 cm^−1^ indicated the successful synthesis of G*_n_*M.

**Figure 2.**
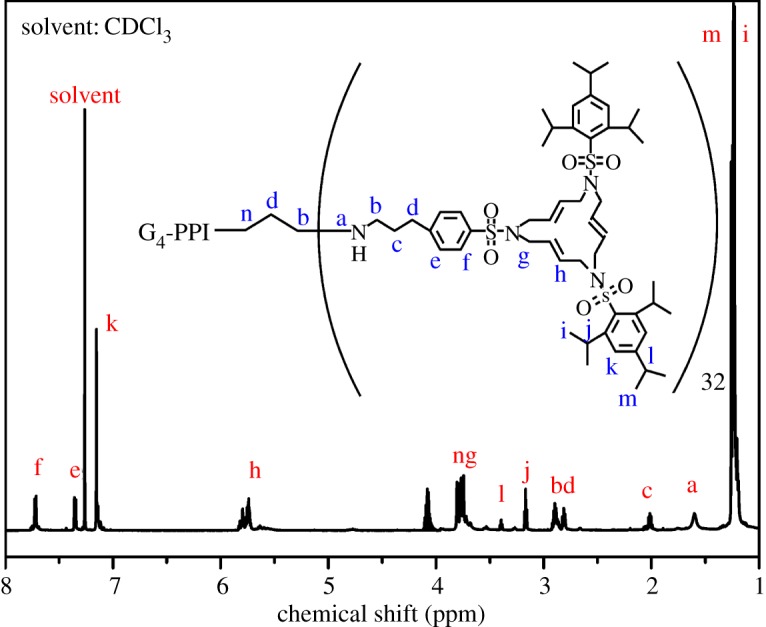
^1^H NMR spectrum of G*_n_*M (*n* = 4) (solvent: CDCl_3_).

Figure 2 shows various kinds of hydrogen in a different chemical environment. The characteristic peaks at 1.20 and 1.18 ppm were attributed to ─C─CH_3_(m) and ─C─CH_3_(i), respectively. And the peaks at 1.60, 1.89, 2.79, 2.88, 3.15, 3.38, 3.76, 3.80, 5.73, 7.15, 7.34, and 7.72 ppm are attributed to ─NH─ (a), ─CH_2_─ (c), ─CH_2_─ (d), ─CH_2_─ (b), ─CH─ (j), ─CH─ (l), ─CH_2_─ (g), ─CH_2_─ (n), ─CH_2_─CH═CH─CH_2_─ (h), aromatic group (k), aromatic group (e), and aromatic group (f). The characteristic peak at 7.26 ppm is attributed to solvent (CDCl_3_). Using the analysis of FTIR in figure 1, the results of ^1^H NMR analysis further proved that G*_n_*M (*n* = 2, 3, 4, 5) are successfully synthesized.

### 3.2. Characterization of G*_n_*M-(Pt*_x_*/Pd_10−*x*_) catalysts

**Figure 3.**
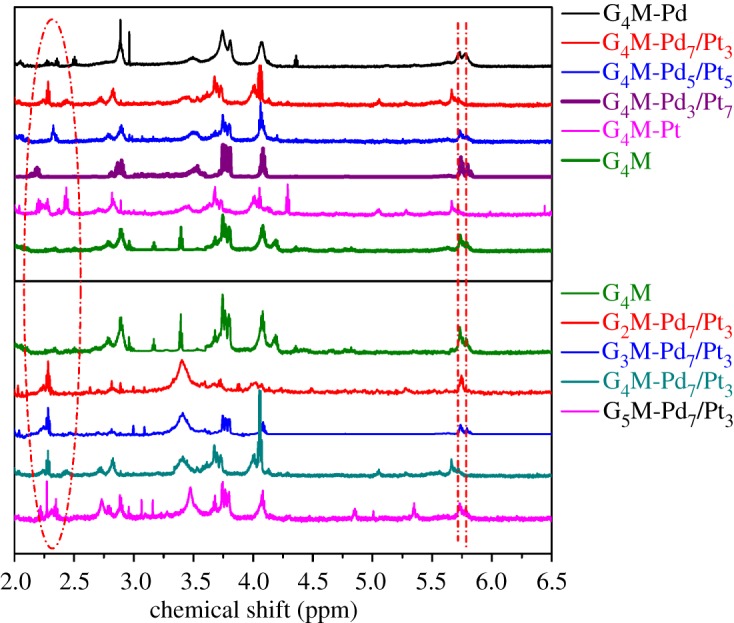
^1^H NMR spectra of G*_n_*M and G*_n_*M-(Pd*_x_*/Pt_10−*x*_) (*n* = 2, 3, 4, 5).

**Figure 4.**
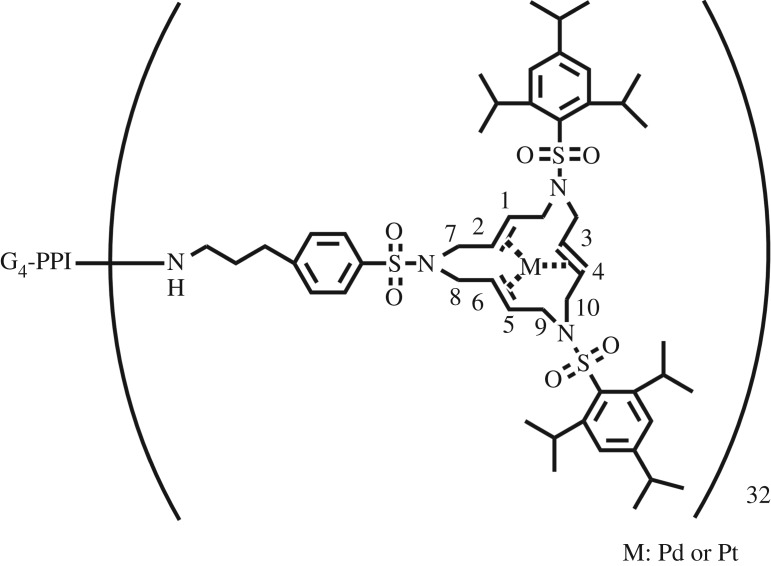
The chemical structure of G*_n_*M-(Pt*_x_*/Pd_10−*x*_) (*n* = 2, 3, 4, 5).

As shown in figure 3, the ^1^H NMR spectra for G_4_M and G*_n_*M-(Pt*_x_*/Pd_10−*x*_) (*n* = 2, 3, 4, 5) are further complicated for the reason that the protons pertaining to the same ethenyl group are not averaged, ascribing to conformational rigidity [24]. These double-bonded protons are in different chemical environments in figure 4; as a consequence, they have various chemical shifts of ^1^H NMR absorptions. The protons of C1–C5, C2–C6, C3–C4 and C7–C8 have similar chemical shifts due to the geometrical symmetry of chemical structure.

The presence of Pd(0) and Pt(0) coordination with the 15-membered triolefinic macrocycle can be concluded from the chemical shifts of ^1^H NMR spectra in table 1. The characteristic peaks at 5.67–5.75 ppm, 5.67–5.79 ppm, 3.67–3.76 ppm and 4.04–4.18 ppm are attributed to protons for C1–C5, C2–C6, C9 and C10 of G_4_M and G*_n_*M-(Pt*_x_*/Pd_10−*x*_), respectively. The peaks located at 2.19–2.52 ppm ascribed to the protons for C3–C4 of G_4_M-(Pt*_x_*/Pd_10−*x*_) (*x* = 0, 3, 5, 7, 1) and G*_n_*M-(Pt_3_/Pd_7_) (*n* = 2, 3, 4, 5) are different from the signals at 5.75 ppm due to the protons for C3–C4 of G_4_M, and the signals of C7 and C8 are shifted from 3.76 to 3.45–3.52 ppm. These chemical shifts arise from the synchronous ligand-exchange reaction between Pt–Pd and G*_n_*M. Hence, it is indicated that G*_n_*M-(Pt*_x_*/Pd_10−*x*_) (*n* = 2, 3, 4, 5) were synthesized successfully by the synchronous ligand-exchange reaction.

**Table 1.** Chemical shifts of ^1^H NMR spectra for G_4_M and G*_n_*M-(Pt*_x_*/Pd_10−*x*_) (*n* = 2, 3, 4, 5).

Positions	1 and 5 (=CH**–**)	2 and 6 (=CH**–**)	3 and 4 (=CH**–**)	7 and 8 (**–**CH_2_**–**)	9 and 10 (**–**CH_2_**–**)
G_4_M	5.75	5.75	5.75	3.76	3.68, 4.18
G_4_M-Pt	5.74	5.74	2.20–2.43	3.45	3.67, 4.04
G_4_M-Pt_7_/Pd_3_	5.73	5.79	2.19	3.52	3.70, 4.08
G_4_M-Pt_5_/Pd_5_	5.67	5.72	2.32	3.51	3.75, 4.07
G_4_M-Pt_3_/Pd_7_	5.67	5.67	2.28–2.45	3.45	3.67, 4.05
G_4_M-Pd	5.72	5.76	2.36–2.52	3.48	3.74, 4.05
G_2_M-Pt_3_/Pd_7_	5.72	5.72	2.27	3.40	3.72, 4.02
G_3_M-Pt_3_/Pd_7_	5.73	5.78	2.28–2.45	3.41	3.76, 4.07
G_4_M-Pt_3_/Pd_7_	5.73	5.79	2.19	3.52	3.70, 4.08
G_5_M-Pt_3_/Pd_7_	5.72	5.77	2.22–2.35	3.45	3.75, 4.08

**Figure 5.**
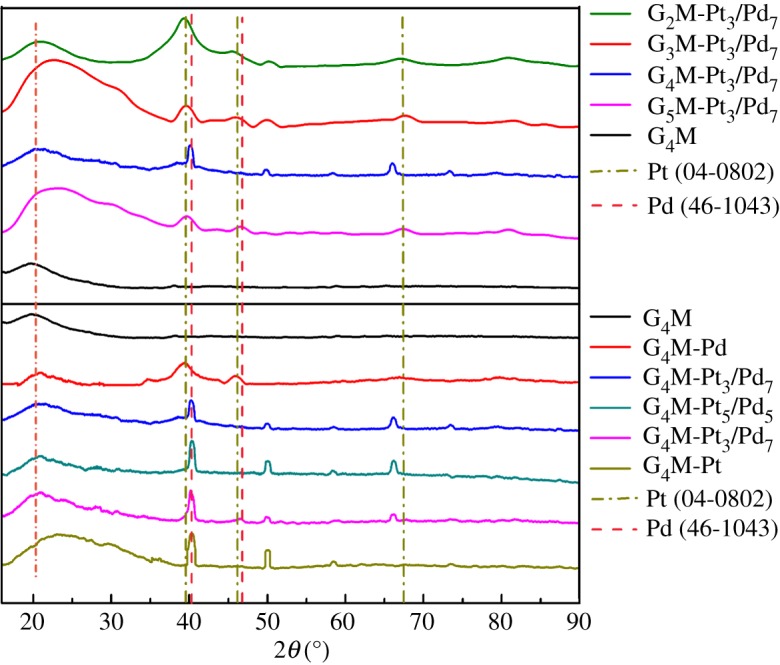
XRD patterns of G_4_M and G*_n_*M-(Pt*_x_*/Pd_10−*x*_) (*n* = 2, 3, 4, 5).

Figure 5 and table 2 show XRD patterns of G_4_M and G*_n_*M-(Pt*_x_*/Pd_10−*x*_). The characteristic peak at 19.97° is assignable to the amorphous peak of G_4_M. The characteristic peaks of G_4_M-Pd at 20.62°, 39.48° and 45.89° are attributed to G_4_M, Pd(1 1 1) and Pd(2 0 0), respectively. The characteristic peaks at 22.62–22.84°, 39.48–40.35°, 45.98–46.66°, 39.44–40.35°, 49.45–49.99° and 66.15–67.89° are ascribed to G*_n_*M (*n* = 4), Pd(1 1 1), Pd(2 0 0), Pt(1 1 1), Pt(2 0 0) and Pt(2 2 0) of G_4_M-(Pt*_x_*/Pd_10−*x*_) (*x* = 7, 5, 3) and G*_n_*M-(Pt_3_/Pd_7_) (2, 3, 4, 5), respectively. Moreover, the characteristic peaks at 23.01°, 40.13° and 49.89° are assignable to G_4_M, Pt(1 1 1) and Pt(2 0 0) of G_4_M-Pt, respectively. Referring to the peaks at 40.12°, 46.66°, 68.12°, 39.76° and 46.24° which are attributed to Pd(1 1 1), Pd(2 0 0), Pt(1 1 1), Pt(2 0 0) and Pt(2 2 0) of standard Pd(JCPDS 46–1043) [25] and Pt(JCPDS 04-0802) [26], respectively, a conclusion can be drawn that G_4_M-(Pt*_x_*/Pd_10−*x*_) and G*_n_*M-(Pt_3_/Pd_7_) (*n* = 2, 3, 4, 5) were synthesized successfully.

**Table 2.** The characteristic peaks for XRD of Pd(0), Pt(0), G_4_M and G*_n_*M-(Pt*_x_*/Pd_10−*x*_) (*n* = 2, 3, 4, 5).

Positions (*h k l*)	G_4_M(°)	Pd (1 1 1) (°)	Pd (2 0 0) (°)	Pt (1 1 1) (°)	Pt (2 0 0) (°)	Pt (2 2 0) (°)
Pt [25]	—	—	—	39.76	46.24	67.45
Pd [26]	—	40.12	46.66	—	—	—
G_4_M	19.97	—	—	—	—	—
G_4_M-Pt	23.01	—	—	40.13	49.89	—
G_4_M-Pt_7_/Pd_3_	20.62	40.35	46.20	40.13	49.45	66.06
G_4_M-Pt_5_/Pd_5_	20.62	40.13	—	40.13	49.67	66.26
G_4_M-Pt_3_/Pd_7_	20.84	40.35	—	40.35	49.67	66.15
G_4_M-Pd	20.62	39.48	45.98	—	—	—
G_2_M-Pt_3_/Pd_7_	21.41	39.44	46.44	39.44	49.68	67.34
G_3_M-Pt_3_/Pd_7_	22.48	39.62	46.45	39.62	49.97	67.89
G_4_M-Pt_3_/Pd_7_	20.84	40.35	—	40.35	49.67	66.15
G_5_M-Pt_3_/Pd_7_	22.54	39.82	46.65	39.82	—	67.52

**Figure 6.**
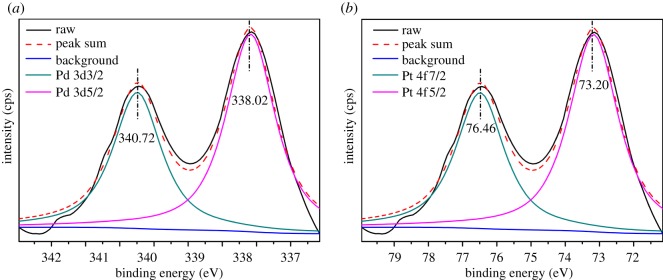
XPS spectra of the Pd 3d (*a*) and Pt 4f (*b*) regions for G_4_M-(Pt_3_/Pd_7_).

XPS was used to confirm the coordination of Pd and Pt with G_4_M. Figure 6 shows the XPS spectra which were adjusted referring to the criterion of the most intense carbon (C1s; binding energy = 284.6 eV). As shown in figure 6*a*, the Pd(3d_3/2_) and Pd(3d_5/2_) peaks lie at 340.72 and 337.82 eV which are different from those peaks (340.63 and 336.15 eV) in G_4_-OH-(Pd) [27]. Meanwhile, figure 6*b* shows the Pt(4f) regions of G_4_M-(Pt_3_/Pd_7_). The Pt(4f_5/2_) and Pt(4f_7/2_) are present at 76.46 and 73.20 eV which were higher than those peaks (72.86 and 76.22 eV) in the G_6_-OH-(Pt_147_) [28]. These differences could also be indicative that the Pt–Pd species of G*_n_*M-(Pt_3_/Pd_7_) are in a bimetallic alloy state.

Table 3 shows the actual quantitative analysis of Pt/Pd atomic ratios for G_2_M-(Pt_3_/Pd_7_) and G_4_M-(Pt_3_/Pd_7_) carried out via ICP-OES. As shown in table 3, the actual Pt/Pd atomic ratios are 0.408 and 0.418 which are close to the experimental ratios (0.43) of G_2_M-(Pt_3_/Pd_7_) and G_4_M-(Pt_3_/Pd_7_), respectively. The decrease of Pt/Pd ratios can be due to the reason that the synchronous ligand-exchange reaction of the precursor Pt is much harder than that of the precursor Pd.

**Table 3.** The actual Pt/Pd atomic ratios of G_2_M-(Pt_3_/Pd_7_) and G_4_M-(Pt_3_/Pd_7_).

samples	Pt	Pd	Pt	Pd	Pt/Pd atomic rations
units	mg kg^−1^	mg kg^−1^	mol kg^−1^	mol kg^−1^	mol mol
G_2_M-Pt_3_/Pd_7_	48 130.89	64 176.47	0.247	0.605	0.408
G_4_M-Pt_3_/Pd_7_	50 373.59	65 486.23	0.258	0.618	0.418

**Figure 7.**
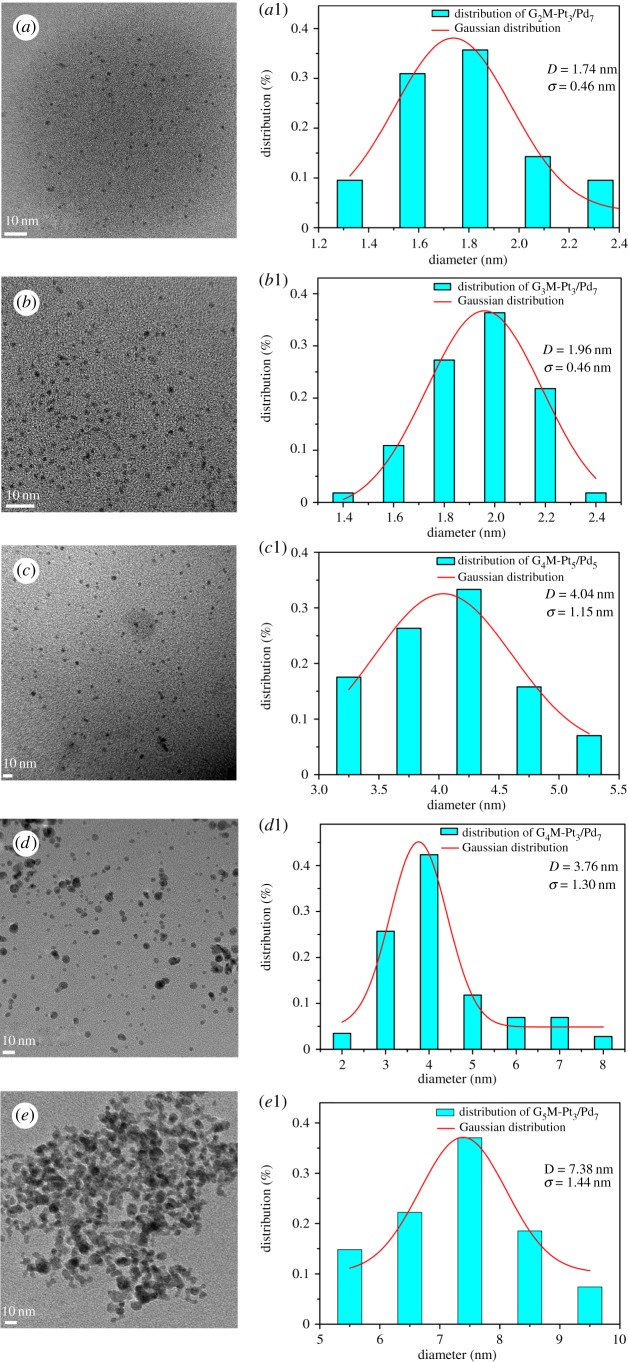
TEM images and core size distribution histograms of (*a*,*a_1_*) G_2_M-(Pt_3_/Pd_7_), (*b*,*b_1_*) G_3_M-(Pt_3_/Pd_7_), (*c*,*c_1_*) G_4_M-(Pt_5_/Pd_5_), (*d*,*d_1_*) G_4_M-(Pt_3_/Pd_7_) and (*e*,*e_1_*) G_5_M-(Pt_3_/Pd_7_).

**Figure 8.**
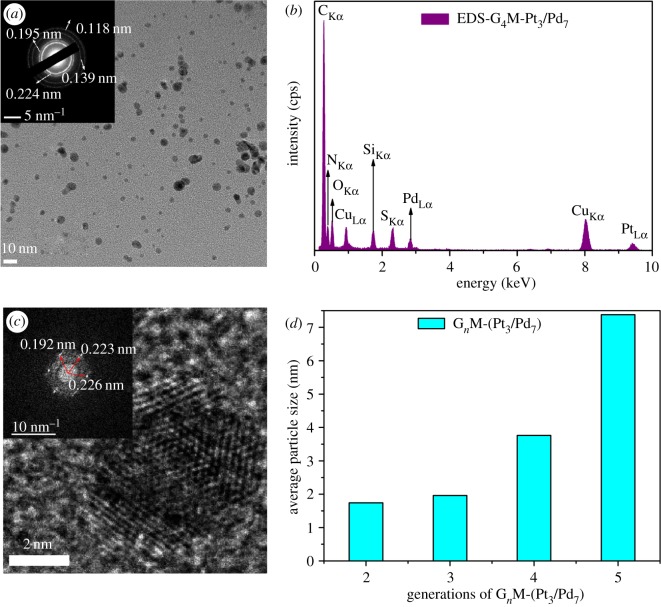
(*a*) TEM micrograph of G_4_M-(Pt_3_/Pd_7_) with the inset showing the electron diffraction pattern of G_4_M-(Pt_3_/Pd_7_) and the calculated *d*-spacing; (*b*) EDS analysis for G_4_M-(Pt_3_/Pd_7_); (*c*) HRTEM micrograph of G_3_M-(Pt_3_/Pd_7_) with the inset showing the FFT pattern for the nanoparticles and the calculated *d*-spacing; and (*d*) the effects of generations of G*_n_*M-(Pt_3_/Pd_7_) (*n* = 2, 3, 4, 5) on the average particle sizes of DSNs.

The representative TEM micrographs of G*_n_*-(Pt_3_/Pd_7_) (*n* = 2, 3, 4, 5) and G_4_-(Pt_5_/Pd_5_) are shown in figure 7. The TEM images demonstrate that the particle size is almost uniform and nearly spherical. As shown in figure 7*c,c_1_* and figure 7*d,d_1_*, the average diameters of G_4_M-(Pt_5_/Pd_5_) and G_4_M-(Pt_3_/Pd_7_) deduced by Gaussian distribution are 4.04 ± 1.15 nm and 3.76 ± 1.30 nm, respectively. Moreover, electron diffraction patterns of G_4_M-(Pt_3_/Pd_7_) in figure 8*a* show that the lattice spaces of 0.224 and 0.139 nm are assigned to Pd(1 1 1) and Pd(2 2 0) combined with the analysis of XRD data [25], respectively. While the lattice spaces of 0.195 and 0.118 nm are attributed to Pt(2 0 0) and Pt(3 1 1) combined with the results of XRD analysis [26], respectively. Using XRD and electron diffraction patterns, it is demonstrated that Pt–Pd species in G_4_M-(Pt_3_/Pd_7_) have an alloy structure. As shown in figure 8*b*, the characteristic X-ray peaks at 2.846 and 9.401 keV are assignable to Pd(La) and Pt(La) [29] in G_4_M-(Pt_3_/Pd_7_), respectively. It is also confirmed that G_4_M-(Pt_3_/Pd_7_) was synthesized successfully.

G*_n_*-(Pt_3_/Pd_7_) (*n* = 2, 3, 5) are also analysed by TEM in figure 7. The Gaussian-fitting average diameters for G_2_M-(Pt_3_/Pd_7_), G_3_M-(Pt_3_/Pd_7_), G_4_M-(Pt_3_/Pd_7_) and G_5_M-(Pt_3_/Pd_7_) are 1.74 ± 0.46, 1.96 ± 0.46, 3.76 ± 1.30, and 7.38 ± 1.44 nm, respectively. As shown in figure 8*d*, it can be indicated that the average sizes of G*_n_*-(Pt_3_/Pd_7_) nanoparticles are positively correlated with the generations of G*_n_*-PPI (*n* = 2, 3, 4, 5). The sizes of G*_n_*-(Pt_3_/Pd_7_) are increased with increasing generations of G*_n_*M. G_5_M has 64 terminated-MACs which is twice as many as G_4_-M and quadruple as many as G_3_M and possesses a lot of coordination sites which can coordinate with much more Pt–Pd metal; therefore, G_5_M-(Pt_3_/Pd_7_) has the largest particle sizes.

Figure 8*c* presents a high-resolution TEM (HRTEM) micrograph of G_3_M-(Pt_3_/Pd_7_) and the correlative diffraction pattern obtained from fast Fourier transform (FFT) analysis. From the inset in figure 8*c*, the characteristic inter-planar spacings (*d*) are observed at 0.192, 0.223 and 0.226 nm in G_3_M-(Pt_3_/Pd_7_) which are close to the standard inter-planar spacings [25,26] Pd-d_200_ = 0.193 nm, Pd-d_111_ = 0.223 nm and Pt-d_111_ = 0.226 nm. It is proved that the G*_n_*-(Pt*_x_*/Pd_10−*x*_) nanoparticles are bimetallic alloy.

### 3.3. Hydrogenation of DA and NBR

**Figure 9.**
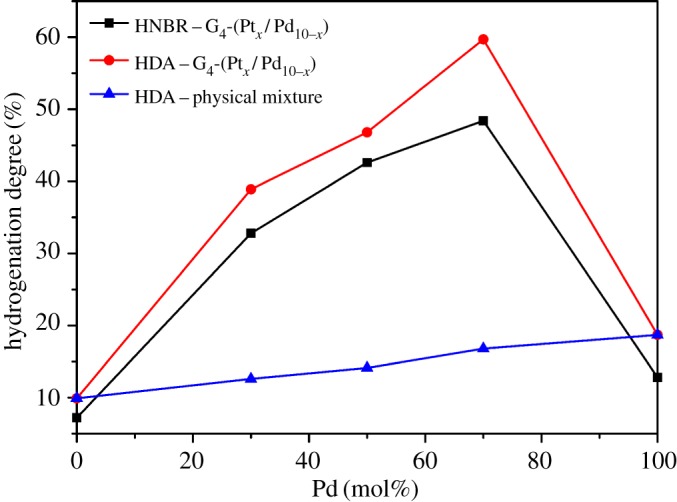
The effects of mole percentages of Pd in G_4_M-(Pt*_x_*/Pd_10−*x*_) (*x* = 0, 3, 5, 7, 10) on the HD of HDA and HNBR. The effects of mole percentages of Pd in the physical mixture of G_4_M-Pt and G_4_M-Pd on the HD of HDA.

To evaluate the catalytic activity and selectivity, G*_n_*-(Pt*_x_*/Pd_10−*x*_) catalysts were applied in the hydrogenation of DA. Figure 9 shows the HD of HDA hydrogenated via G_4_-(Pt*_x_*/Pd_10−*x*_) (*x* = 0, 3, 5, 7, 10) and the physical mixture of G_4_M-Pt and G_4_M-Pd. HDA catalysed by bimetallic DSN catalysts has a higher HD than that catalysed by the physical mixture of monometallic DSN catalysts with equal components of Pd and Pt. The maximum HD is 59.7% catalysed by G_4_-(Pt_3_/Pd_7_) which is much higher than that of 18.7% catalysed by G_4_M-Pd only. The improved catalytic activity of bimetallic DSNs indicates that there are alloying and bimetallic synergistic electronic effects [30] in the G*_n_*-(Pt_3_/Pd_7_) nanoparticles. A synergetic effect can be caused by a structure effect and chemical effect [31]. Structure synergism indicates the increasing number of new active sites in bimetallic DSNs. It may lead to a greater dispersion of the two metals than in the monometallic catalysts; furthermore, it may have mutual effects between two metals. Chemical synergism means that the bimetallic catalyst contains new sites not present in the monometallic catalysts, which will lead to different activities of the reaction intermediates over the monometallic and bimetallic catalysts.

**Figure 10.**
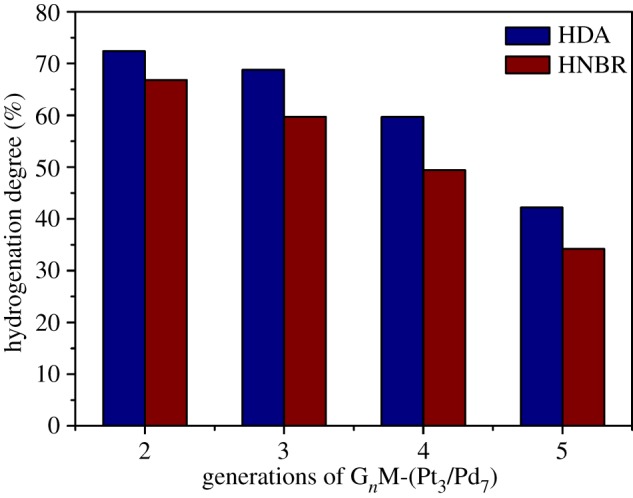
The effects of generations of G*_n_*-(Pt_3_/Pd_7_) (*n* = 2, 3, 4, 5) on the HD of HDA and HNBR.

Figure 9 also shows catalytic hydrogenation effects for DA and NBR using G_4_-(Pt*_x_*/Pd_10−*x*_) (*x* = 0, 3, 5, 7, 10) catalysts. From the hydrogenated curves in figure 9 for HDA and HNBR, it can be seen that the HD for HDA is higher than that of HNBR with the equal composition of G*_n_*-(Pt*_x_*/Pd_10−*x*_). The maximum HD for HDA and HNBR were 59.7% and 48.4%, respectively. As shown in figure 10, the HD for HDA is also higher than that of HNBR catalysed by the same generation of G*_n_*-(Pt_3_/Pd_7_). The maximum HD for HDA and HNBR were 72.4% and 66.8%, respectively. It may be due to the significant difference of molecular mass of DA and NBR: DA is classified as an oligomer (*M*_w _= 300.2), while NBR is classified as macromolecular (*M*_w_ ≈ 350 000). The viscosity of NBR solution is much higher than that of DA. Therefore, it is the reason that the HD of HNBR is lower than that of HDA.

Figure 10 presents the HD of NBR and HDA catalysed by G*_n_*-(Pt_3_/Pd_7_) (*n* = 2, 3, 4, 5). It can be seen that the HD of G*_n_*-(Pt_3_/Pd_7_) can be fitted with a remarkable negative correlation with the incremental generations of G*_n_*-(Pt_3_/Pd_7_). The maximum HD for HDA and HNBR catalysed by G_2_-(Pt_3_/Pd_7_) are 72.4% and 66.8%, respectively, while the minimum HD for HDA and HNBR catalysed by G_5_-(Pt_3_/Pd_7_) are 44.2% and 34.2%, respectively. The average particle sizes of G*_n_*-(Pt*_x_*/Pd_10−*x*_) were increased with increasing generations of G*_n_*M. Nano-catalysts with smaller sizes, having a higher specific surface area, can enhance the dispersity of active sites, which is beneficial to absorb reactants and H_2_ on the surface of catalysts [32]. As a consequence, G*_n_*-(Pt*_x_*/Pd_10−*x*_) with smallest sizes have the highest catalytic activity for unsaturated compounds.

## 4. Conclusion

G_*n*_-PPI (*n* = 2, 3, 4, 5) terminated with N-containing 15-membered triolefinic macrocycle have been synthesized. The novel mono-dispersed Pt–Pd bimetallic nanoparticles encapsulated by G*_n_*M (*n* = 2, 3, 4, 5) dendrimers have been prepared by the synchronous ligand-exchange reaction of Pt(PPh_3_)_4_ and Pd(PPh_3_)_4_ successfully. The average diameters of G*_n_*-(Pt_3_/Pd_7_) (from 1.74 to 7.38 nm) are increased with increasing generations of G*_n_*M. The ^1^H NMR, XRD, XPS, EDS and HRTEM analyses have also demonstrated that alloy-structure Pt–Pd bimetallic nanoparticles were synthesized successfully. In contrast to the HD of HDA catalysed by physical mixture of monometallic DSNs, the G*_n_*-(Pt*_x_*/Pd_10−*x*_) DSNs have exhibited a high catalytic activity, alloying and bimetallic synergistic electronic effect as hydrogenated catalysts, the highest activity being achieved with a Pt/Pd ratio of 3 : 7. The catalytic activities for DA are higher than those for NBR. The catalytic activities for HDA and HNBR present negative correlation with the incremental generations of G*_n_*-(Pt_3_/Pd_7_), the highest activity being obtained with G_2_-(Pt_3_/Pd_7_).

## Data accessibility

Our data are deposited at Dryad: http://dx.doi.org/10.5061/dryad.7j4t8 [33].
